# Recurrent CNTN1 antibody-positive nodopathy: a case report and literature review

**DOI:** 10.3389/fimmu.2024.1368487

**Published:** 2024-05-23

**Authors:** Min Zhao, Guixian Chen, Shuguang Li, Xiaojun Li, Haoxuan Chen, Zhenzhen Lou, Huiying Ouyang, Yibo Zhan, Chenghao Du, Yuanqi Zhao

**Affiliations:** ^1^ The Second Affiliated Hospital of Guangzhou University of Chinese Medicine, Guangdong Provincial Hospital of Chinese Medicine, Guangzhou, Guangdong, China; ^2^ State Key Laboratory of Dampness Syndrome of Chinese Medicine, The Second Affiliated Hospital of Guangzhou University of Chinese Medicine, Guangzhou, Guangdong, China; ^3^ The Second School of Clinical Medicine, Guangzhou University of Chinese Medicine, Guangzhou, Guangdong, China; ^4^ School of International Relations, National University of Defense Technology, Nanjing, China

**Keywords:** contactin-1, nodopathy, CIDP, tremor, ataxia

## Abstract

**Background:**

Contactin-1 (CNTN1) antibody-positive nodopathy is rare and exhibits distinct clinical symptoms such as tremors and ataxia. However, the mechanisms of these symptoms and the characteristics of the cerebral spinal fluid (CSF) remain unknown.

**Case presentation:**

Here, we report a case of recurrent CNTN1 antibody-positive nodopathy. Initially, a 45-year-old woman experiencing numbness in the upper limbs and weakness in the lower limbs was diagnosed with chronic inflammatory demyelinating polyradiculoneuropathy (CIDP). Eleven years later, her symptoms worsened, and she began to experience tremors and ataxia. Tests for serum CNTN1, GT1a, and GQ1b antibodies returned positive. Subsequently, she was diagnosed with CNTN1 antibody-positive nodopathy and underwent plasmapheresis therapy, although the treatment’s efficacy was limited. To gain a deeper understanding of the disease, we conducted a comprehensive literature review, identifying 52 cases of CNTN1 antibody-positive nodopathy to date, with a tremor prevalence of 26.9%. Additionally, we found that the average CSF protein level in CNTN1 antibody-positive nodopathy was 2.57 g/L, with 87% of patients exhibiting a CSF protein level above 1.5 g/L.

**Conclusion:**

We present a rare case of recurrent CNTN1 antibody-positive nodopathy. Our findings indicate a high prevalence of tremor (26.9%) and elevated CSF protein levels among patients with CNTN1 antibody-positive nodopathy.

## 1 Introduction

**Figure 1:**
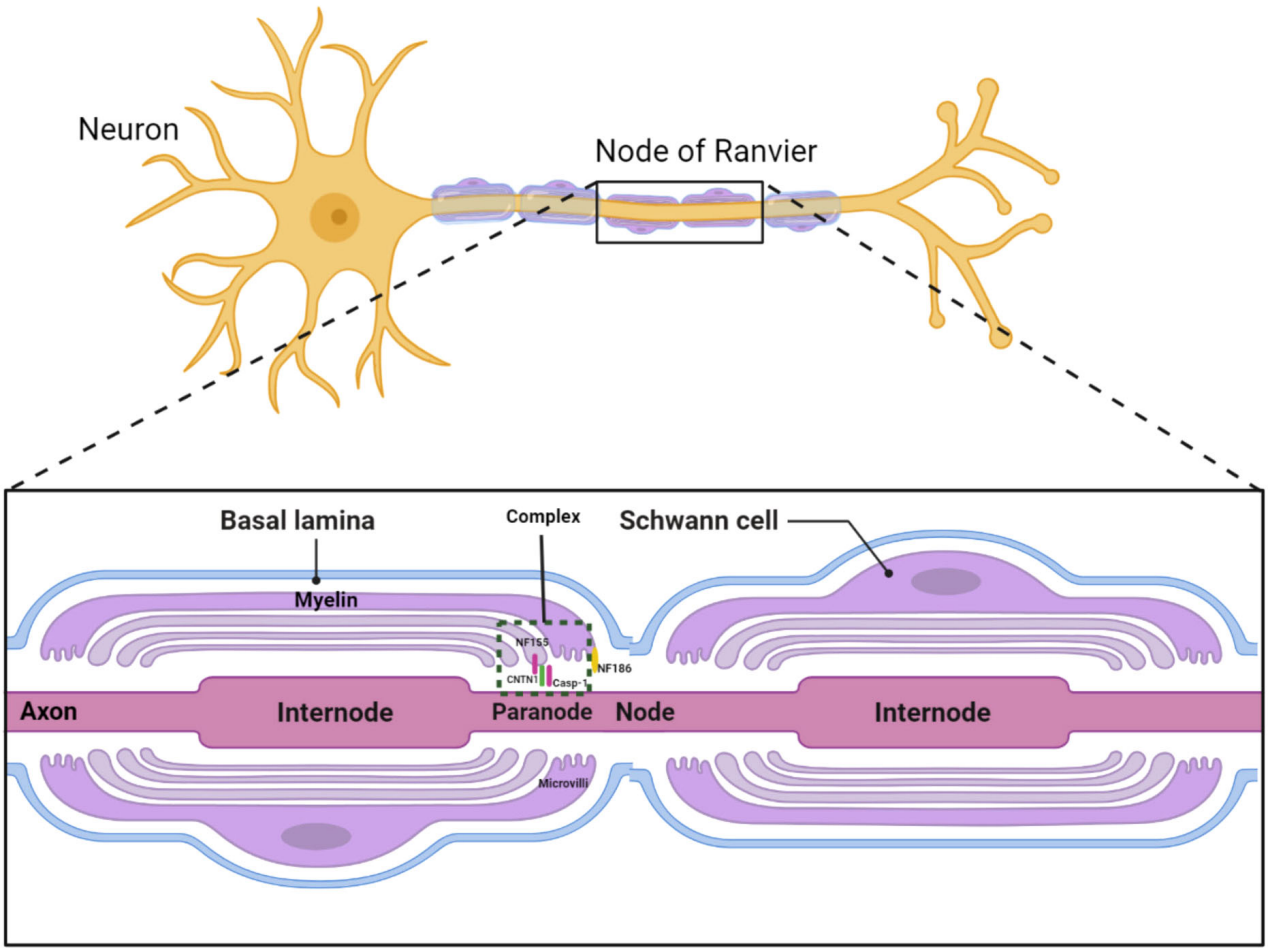
Diagram of the antibody complex in the Ranvier node region.

Chronic inflammatory demyelinating polyradiculoneuropathy (CIDP) is the most common form of chronic inflammatory neuropathy, characterized by segmental demyelination. Beyond demyelinating or axonal damage, recent studies introduced a concept termed “nodopathy”, referring to microstructural alterations limited to the nodal and paranodal regions, potentially leading to significant nerve dysfunction (1, 2). In patients with CNTN1 antibody positivity, paranodal destruction and axo-glial disjunction have been documented. The cell adhesion molecule neurofascin-186 (NF186) anchors voltage-gated sodium channels at the node, while the glial protein neurofascin-155 (NF155), along with axonal proteins contactin-1 (CNTN1) and contactin-associated protein-1 (CASPR1), constitutes an axoglial complex in the paranodal region (**Figure 1**) (3). Therefore, CIDP cases with NF155 and CNTN1 antibodies might exhibit similar pathophysiology and clinical manifestations, including distal dominant symptoms and axonal degeneration, differing from the typical CIDP symptoms of proximal and distal muscle weakness with less pronounced axonal degeneration (4).

Approximately 10% of CIDP patients exhibit autoantibodies against nodal and paranodal proteins, with anti-NF155 antibodies present in 4%–18% of patients and antibodies against CNTN1, CASPR1, and the CNTN1-CASPR1 complex reported in 1%–7% of patients (5).

Querol et al. described the characteristics of neuropathy with CNTN1 antibody as including older age at onset, Guillain–Barre syndrome-like acute onset of weakness, sensory ataxia, and early axonal involvement (6), noting a higher prevalence of tremor among those with positive antibodies. The response to corticosteroids and intravenous immune globulin (IVIG) was suboptimal (3).

Given the distinct clinical presentations between typical CIDP and nodopathy, their pathogenic mechanisms might be different. Here, we report a case of recurrent CNTN1 antibody-positive nodopathy and discuss the possible mechanisms of tremor and ataxia, and the CSF characteristics, to deepen our understanding of the disease.

## 2 Case presentation

### 2.1 First episode

#### 2.1.1 Medical history and physical examination

On 15 December 2010, a 45-year-old woman was admitted to our hospital with primary complaints of numbness in the upper limbs and weakness in the lower limbs, persisting for 5 months. Neurological examination revealed that the Medical Research Council (MRC) grade (**Table 1**) for the upper limbs was normal, while the MRC grade for the lower limbs was grade 2. Deep-tendon reflexes across all four limbs were weak. She had a medical history of nephrotic syndrome but no family history of hereditary conditions or infectious diseases.

**Table 1:** Medical research council (MRC) scale for muscle examination.

Score for each movement	
0—No visible contraction	
1—Visible muscle contraction, but no limb movement	
2—Active movement, but not against gravity	
3—Active movement against gravity	
4—Active movement against gravity and resistance	
5—Active movement against full resistance	

#### 2.1.2 Auxiliary examination

Electromyography (EMG) results indicated demyelinating alterations and axonal damage in motor and sensory nerves, along with F-wave prolongation (**Table 2**). CSF analysis revealed a protein concentration of 1,310 mg/L and a white blood cell count of 2 × 10^6^/L.

**Table 2:** The results of EEG of two episodes.

Nerve	Test Time	
	First Episode(Left/Right)	Second Episode(Left/Right)
Median Nerve		
CMAP (mV, wrist)	5.1/2.6	1.5/1.2
CMAP (mV, elbow)	4.5/1.7	0.7/0.5
DML (ms, wrist to elbow)	8.4/13.2	11.1/16.3
MCV (m/s, wrist to elbow)	36/29	18/13
SCV (m/s, little finger to wrist)	NR	NR
SNAP (μV, finger to wrist)	NR	NR
F - Latency(ms)	NR	NR
F%	0	0
Ulnar Nerve		
CMAP (mV, wrist)	5.3/7.2	0.8/0.7
CMAP (mV, below elbow)	4.2/4,5	1.3/NR
CMAP (mV, above elbow)	4.1/4.2	0.3/NR
DML (ms, wrist)	7.8/7.5	9.7/7.2
MCV (m/s, wrist to below elbow)	28/28	13/NR
MCV (m/s, wrist to above elbow)	34/42	16/NR
SCV (m/s, little finger to wrist)	NR	NR
SNAP (μV, finger to wrist)	NR	NR
F-Latency(ms)	54/47.6	NR/42
F%	68.8/76.9	NR/25
Superficial Peroneal Nerve		
CMAP (mV)	NR	NR
DML (ms)	NR	NR
MCV (m/s)	NR	NR
SCV (m/s)	NR	NR
SNAP (μV)	NR	NR
F-Latency (ms)	ND	ND
F%	ND	ND
Sural Nerve		
CMAP (mV)	NR	NR
DML (ms)	NR	NR
MCV (m/s)	NR	NR
SCV (m/s)	NR	NR
SNAP (μV)	NR	NR
F-Latency(ms)	ND	ND
F%	ND	ND
Tibial Nerve		
CMAP (mV)	NR	NR
DML (ms)	NR	NR
MCV (m/s)	NR	NR
SCV (m/s)	NR	NR
SNAP (μV)	NR	NR
F-Latency(ms)	NR	NR
F%	NR	NR

CMAP, compound motor active potential; DML, distal motor latency; MCV, motor nerve conduction velocity; SCV, sensory nerve conduction velocity; SNAP, sensory nerve active potential; EEG, electromyography; ND, not examined; NR, not elicited.

#### 2.1.3 Diagnosis and treatment

Following the European Federation of Neurological Societies/Peripheral Nerve Society Guideline on the management of chronic inflammatory demyelinating polyradiculoneuropathy (2010) (7), the diagnosis of CIDP was considered. After treatment with intravenous prednisolone, the patient’s symptoms improved, leading to her discharge.

### 2.2 Second episode

#### 2.2.1 Medical history and physical examination

Eleven years later, the same patient was readmitted to our hospital with numbness and weakness in all four limbs, accompanied by tremor. She required assistance to walk and could not use chopsticks or a pen.

The physical examination revealed bilateral upper limb tremors (Video 1). The MRC grade for the proximal strength in both upper and lower limbs was grade 4, while the distal strength in all four limbs was grade 2. There was noticeable sensory disturbance, including deep sensory disturbances, in the extremities. Tendon reflexes in all four limbs were absent. The patient was unable to perform the finger–nose test and the heel–knee–tibia test successfully. The Babinski sign was absent.

#### 2.2.2 Auxiliary examination

No abnormalities were detected in blood routine tests, coagulation function, interleukin 6 levels, thyroid function, vasculitis testing, or immunoelectrophoresis (blood and urine). CSF analysis showed a protein concentration of 1,344 mg/L and a white blood cell count of 5 × 10^6^/L. The serum immunoglobulin G4 level was 1,283.4 (reference value, 39.2–864).

This time, testing for peripheral ganglioside antibodies and antiparanodal antibodies was conducted. The serum CNTN1 antibody [confirmed by Cytometric Bead Assay (CBA)] tested positive with a titer of 1:1,000+. Additionally, anti-GT1a and anti-GQ1b IgG antibodies in serum were also positive. All tests were performed at Jiangsu Simcere Diagnostic Laboratory (Jiangsu Simcere Diagnostics Co, Ltd, Nanjing 210002, China). Brain MRI, brachial plexus, and lumbosacral MRI showed no evident abnormalities. Electromyography (EMG) indicated worsened demyelinating and axonal damage (**Table 2**).

#### 2.2.3 Diagnosis and treatment

The patient was diagnosed with CNTN1 antibody-positive nodopathy. Following plasmapheresis therapy, the MRC grade for distal strength in all four limbs improved to 3. However, she remained unable to walk without assistance. Two months post-discharge, no significant improvement was observed.

## 3 Discussions

Autoantibodies targeting the molecular components of the node of Ranvier proteins, such as neurofascin, contactin-1, and Caspr, have recently been identified in CIDP. The latest guidelines delineate antibody-positive diseases associated with paranodopathy as autoimmune nodopathies (8). Querol first identified the CNTN1 antibody as the target antigen in three patients diagnosed with CIDP (6). These individuals exhibited an aggressive disease phenotype characterized by acute onset, predominantly motor involvement, older age at onset, and, notably, a poor response to IVIg. Subsequently, it was discovered that CNTN1 antibodies were predominantly IgG4, potentially explaining the suboptimal responses to IVIg (9). Diverging from typical CIDP, CNTN1 antibody-positive nodopathy presents distinct clinical features: (1) older age at onset, (2) subacute or chronic onset with progressive development, (3) common occurrences of ataxia and tremor, and (4) frequent deep sensation disturbances (10). Additionally, total cerebrospinal fluid protein levels were elevated. Herein, we report a case of recurrent CNTN1 antibody-positive nodopathy and discuss the possible mechanisms behind tremor and ataxia and the CSF characteristics.

### 3.1 Tremor and ataxia could be a characteristic symptom of CNTN1 antibody positive nodopathy

Traditionally, tremor and ataxia have been distinct characteristics of NF155 antibody-positive paranodopathy (5, 11, 12). Querol observed that the NF155 antibody binds to the cerebellum, particularly cerebellar neurons, potentially explaining the presence of tremor and ataxia (13).

Initially, Querol identified CNTN1 antibodies in CIDP patients, although none of the three cases exhibited tremor (6). Later, Yumako’s cohort, which included 13 CNTN1 antibody-positive patients out of 533 CIDP patients, reported two instances of tremor (14). Doppler’s cohort described three patients experiencing tremor, both rest and action types. Given the specificity of tremor as a symptom, it may serve as a differential marker between typical CIDP and nodopathy. Further investigations by Doppler and colleagues demonstrated that sera from patients with positive CNTN1 antibodies reacted to the molecular and granular layers of the cerebellum, offering a possible explanation for tremor (10). A review of the literature revealed 52 cases of CNTN1 antibody-positive nodopathy (**Table 3**), with 14 patients (26.9%) exhibiting tremor, suggesting it as a characteristic symptom of CNTN1-positive nodopathy.

**Table 3:** Cases of CNTN1 antibody nodopathy.

Year	Author	Patient	Age of onset	Sex	Tremor	CSF protein level (g/L)	CSF≥1.5g/L	CSF white cell count
2013	Luis Querol (6)	1	77	F	No	1.62	Yes	1
		2	60	M	No	2.46	Yes	5
		3	76	F	No	3.75	Yes	13
2015	Yumako Miura (14)	4	75	M	No	2.61	Yes	4
		5	81	M	No	1.69	Yes	10
		6	63	M	Yes	3.8	Yes	NA
		7	58	M	No	0.79	No	1
		8	33	F	No	1.02	No	6
		9	71	M	No	6.93	Yes	6
		10	59	F	No	1.82	Yes	4
		11	70	M	No	3.85	Yes	6
		12	47	F	No	1.5	Yes	NA
		13	60	M	No	1.92	Yes	2
		14	63	M	No	2.8	Yes	2
		15	72	M	No	1.85	Yes	2
		16	36	M	Yes	1.59	Yes	0
2015	Kathrin Doppler (10)	17	NA	NA	No	NA	NA	NA
		18	NA	NA	Yes	NA	NA	NA
		19	NA	NA	Yes	NA	NA	NA
		20	NA	NA	Yes	NA	NA	NA
2017	Emilien Demont (15)	21	NA	NA	NA	NA	NA	NA
		22	NA	NA	NA	NA	NA	NA
2017	Hsin-Pin Lin (16)	23	20	M	No	NA	NA	NA
2020	Zhu Ju (17)	24	74	M	Yes	1.77 and	Yes	NA and 4
						2.03		
2020	Cortese Andrea (12)	25–27	58 average	M	1 Yes	1.48 average	NA	NA
					2 No			
2021	Ying Huang (18)	28	57	M		1.65	Yes	7
2022	Qinzhou Wang (19)	29	50	M	Yes	3.45	Yes	0
		30	54	M	Yes	5.22	Yes	0
2022	Simon Rinaldi (20)	31	56	M	Yes	0.32	No	<5
		32	49	M	No	2.1	Yes	<5
		33	39	M	Yes	0.24	No	<5
		34	50	M	No	2.8	Yes	28
		35	79	M	No	2.4	Yes	<5
		36	74	M	No	1.5	Yes	<5
		37	62	M	No	2.08	Yes	14
2022	Eduardo Nobile-Orazio (21)	38–41	52.5 average	75% M	No	1.865 average	NA	NA
2023	Maarten J Titulaer (22)	42–47	72 average	17% M	3 Yes	1.04 average	NA	NA
					3 No			
2023	Jing Zhang (23)	48	62	M	Yes	6.18	Yes	6
2023	Lingchao Meng (24)	49	60	M	No	NA	NA	NA
		50	46	F	No	1.96	Yes	8
		51	14	M	No	2.84	Yes	1
		52	33	F	No	4.8	Yes	9

F, female; M, male; NA, unknown.

Tremor is recognized as an accompanying feature of inflammatory-mediated peripheral neuropathies such as immunoglobulin M paraproteinemic neuropathy (25) and CIDP (26, 27). Previous studies have not established a direct relationship between tremor and conduction velocity (28). The speculated mechanism involves central compensation by the cerebellum for delays induced by peripheral neuropathy (29). Moreover, tremor can manifest as a rare symptom of GBS, particularly in specific variants like Miller–Fisher syndrome, where the mechanism is linked to cerebellar function and impaired sensorimotor feedback (30). In the case discussed, the patient did not exhibit tremors during the initial episode nor were ganglioside or paranodal antibodies tested. However, 11 years later, the patient’s symptoms, including tremor and ataxia, worsened. Tests indicated positivity for GT1a, GQ1b, and CNTN1 antibodies. It remains uncertain whether this signifies a change in antibody type or a CNTN1 subtype.

Ataxia has been identified as a common symptom of CNTN1 antibody-positive nodopathy. The mechanisms might involve injury to the Ranvier nodes (31) and the dorsal root ganglia (14, 32). Initially, the CNTN1 and CASPR1 dimer is crucial for maintaining the axon–glial linkage at the paranode and neuronal conduction at the Ranvier nodes (33). *In vitro* studies have shown that CNTN1 antibodies can disrupt the interaction between the CNTN1/Caspr1 complex and NF155, altering the structure of the Ranvier node region and contributing to symptoms of CNTN1-related CIDP (34). Second, Yumako Miura and colleagues found that CNTN1 is widely expressed in dorsal root ganglion neurons (14). Additionally, the dorsal root ganglia’s blood–nerve barrier is more permeable, allowing CNTN1 antibodies to infiltrate sensory neurons and axons, which may explain sensory ataxia (35). The patient being double-positive for CNTN1 and GT1a and GQ1b antibodies complicates the diagnosis. Given that ataxia is a prevalent sign of Miller–Fisher syndrome, associated with GT1a and GQ1b antibodies (36), it raises the question of whether the ataxia in this case is caused by the CNTN1 antibody or antiganglioside antibodies (GT1a and GQ1b antibodies). At present, the mechanism of tremor and ataxia in CNTN1 antibody-positive nodopathy is still not clear, and further research is needed.

### 3.2 CSF protein is obviously elevated among CNTN1 antibody-positive nodopathy

In the case of CNTN1 antibody-positive nodopathy, CSF data for eight patients were unavailable. The average CSF protein levels reported were 1.865 g/L in Orazio’s study (21), 1.48 g/L in Cortese’s study (12), and 1.04 g/L in Titulaer’s study (22). Excluding these three studies and the eight patients without CSF data, the average CSF protein level for the remaining 31 patients was 2.57 g/L, with 27 patients (87%) exhibiting a CSF protein level higher than 1.5 g/L (**Table 3**).

Albuminocytological dissociation, characterized by increased protein levels (>0.45 g/L) in the absence of an elevated white cell count (<50 cells/μL), is a hallmark of GBS (37). High CSF protein levels (>0.45 g/L) have been linked to the demyelinating subtype and proximal or global muscle weakness in patients with GBS. Moreover, a higher CSF protein level has been independently associated with an increased likelihood of poor outcomes in patients with brainstem encephalitis (38). The specific CSF characteristics of paranodopathy, including the relationship between CSF protein levels and prognosis, remain unclear. Through literature review, we identified the CSF characteristic of CNTN1 antibody-positive nodopathy as notably high protein levels, which could serve as a differential diagnostic marker. Future cohort studies should further explore the CSF characteristics of paranodopathy and their correlation with prognosis.

### 3.3 CNTN1 antibody-positive nodopathy with antiganglioside antibodies

Gangliosides, essential components of peripheral nerves, consist of ceramide bonded to one or more sugars, incorporating sialic acid attached to an oligosaccharide core. Gangliosides are categorized into GM1, GD1a, GT1a, and GQ1b, based on the number and placement of sialic acids. Previous studies identified that antibodies against GM1 and GD1a were linked to acute motor axonal neuropathy (36). The GQ1b antibody, which cross-reacts with the GT1a antibody, has been closely associated with Miller–Fisher syndrome and brainstem encephalitis, characterized by ophthalmoplegia, ataxia, and impaired consciousness (39–41). Theoretically, GQ1b antibodies could explain the sensory ataxia observed in our case. These antibody target molecules have been seldom detected in CIDP patients, and no studies have yet explored the relationship between antiganglioside antibodies and nodopathy. One investigation reported a 12% prevalence of GM1 IgM antibodies among CIDP patients, significantly lower than in multifocal motor neuropathy (60%) (42). A cohort study involving Chinese GBS and CIDP patients identified GM1 as the target antigen in CIDP patients’ sera, noting that the IgM type was more prevalent than IgG in these patients. To our knowledge, this case marks the first instance of CIDP concurrently positive for both CNTN1 antibody and antiganglioside antibodies (anti-GT1a and anti-GQ1b antibodies). The interplay between nodopathy antibodies and antiganglioside antibodies in CIDP and its symptomatic manifestations warrants further investigation.

## 4 Conclusions

We have reported a rare case of recurrent CNTN1 antibody-positive nodopathy, also positive for GT1a and GQ1b antibodies. Our findings highlight a tremor prevalence of 26.9% among CNTN1 antibody-positive nodopathy cases and suggest that the CSF protein level in such cases may be significantly elevated compared to typical chronic inflammatory demyelinating polyradiculoneuropathy (CIDP).

## Data availability statement

The raw data supporting the conclusions of this article will be made available by the authors, without undue reservation.

## Ethics statement

The studies involving humans were approved by Ethics Committee of Guangdong Provincial Hospital of Chinese Medicine. The studies were conducted in accordance with the local legislation and institutional requirements. The participants provided their written informed consent to participate in this study. Written informed consent was obtained from the individual(s) for the publication of any potentially identifiable images or data included in this article. Written informed consent was obtained from the participant/patient(s) for the publication of this case report.

## Author contributions

MZ: Writing – review & editing, Writing – original draft, Formal Analysis, Data curation, Conceptualization. GC: Writing – review & editing, Writing – original draft, Methodology, Data curation. SL: Writing – review & editing, Data curation. XL: Writing – review & editing, Investigation. HC: Writing – review & editing, Formal Analysis, Data curation. ZL: Writing – review & editing, Investigation. HO: Writing – review & editing, Investigation. YBZ: Writing – review & editing, Investigation. CD: Writing – review & editing. YQZ: Writing – review & editing, Project administration, Funding acquisition, Conceptualization.
